# Reproductive health and healthcare experiences in autistic and non-autistic individuals assigned female at birth

**DOI:** 10.1177/17455057261465645

**Published:** 2026-07-07

**Authors:** Lili Bokor, Francesca Happé, Elizabeth Weir, Donghao Lu, Gavin R. Stewart, Miriam I. Martini, Mark J. Taylor

**Affiliations:** 1 Department of Forensic and Neurodevelopmental Sciences, Institute of Psychiatry, Psychology and Neuroscience, King’s College London, London, UK 34426; 2 Social, Genetic and Developmental Psychiatry Centre, King's College London, London, UK 47957; 3 151964 Autism Research Centre, University of Cambridge, Cambridge, UK; 4 193414 Institute of Environmental Medicine, Karolinska Institutet, Stockholm, Sweden; 5 Department of Medical Epidemiology and Biostatistics, 211741 Karolinska Institutet, Stockholm, Sweden

**Keywords:** autism, cross-sectional, reproductive health, gynecological conditions, healthcare inequalities, menstrual health

## Abstract

**Background:**

Despite increased recognition of autism in women and girls, their reproductive health remains underexplored. Understanding reproductive health burden and healthcare experiences is essential to identifying barriers and improving support for conditions that can impact quality of life.

**Objectives:**

Investigate reproductive health and healthcare experiences among autistic compared to non-autistic individuals assigned female at birth (AFAB).

**Design:**

We conducted a cross-sectional online survey in the UK (April 2024–July 2025) among individuals AFAB aged 18–40 years recruited via convenience-sampling from autism networks, social media, and Prolific.

**Methods:**

In total, 311 participants were included (165 self-reported autistic [*M*=31.1 years, *SD*=6.2], 146 non-autistic [*M*=30.6 years, *SD*=5.5]). The survey, developed with input from autistic people, covered reproductive health conditions, knowledge and management of reproductive health, and reproductive healthcare experiences. Group differences were analysed using logistic regressions, chi-squared and Wilcoxon rank-sum tests. Healthcare inequality (HIE) scores were calculated overall and for five subdomains as composite of negative reproductive healthcare experiences. Associations between autism and HIE were examined using logistic regression.

**Results:**

Autistic participants reported more reproductive health conditions (44% vs. 28%) and symptoms (95% vs. 84%) than non-autistic participants. Age-adjusted regression models indicated higher odds for any condition (OR=1.96[1.21-3.17], *p* <.01) and any symptom (OR=3.23[1.44-7.25], *p* <.01) with OR for specific conditions/symptoms ranging from OR=1.09[0.57-2.09], *p*=.799 to OR=3.97[2.43-6.50], *p* <.001. Adjusting for other neurodivergence attenuated estimates; however, the overall associations for any symptom remained statistically significant (*p* <.05). Autistic participants were more likely to report irregular menstrual cycles, menstrual cycle–related mental health and sensory experiences changes and poorer reproductive health knowledge and management (all *p* <.001). HIE scores overall and across subcategories were higher among autistic individuals. Autism diagnosis was associated with higher overall HIE scores (OR=2.86[2.37-3.45], *p* <.001) and domain specific HIE scores (OR_range_ = 1.81[1.49-2.22]–5.31[3.56-8.13], *p* <.001).

**Conclusion:**

Autistic individuals AFAB face increased reproductive health burden, greater difficulty managing their reproductive health, and significant healthcare inequities. Tailored education and individualized service adjustments are essential for equitable reproductive care in autistic individuals AFAB.

## Plain language summary

Autistic individuals assigned female at birth (AFAB) often have unique health needs, but little is known about their experiences with reproductive health and healthcare. This study looked at the reproductive health and healthcare experiences of autistic AFAB individuals compared with non-autistic AFAB individuals. We conducted an online survey in the UK with 311 participants aged 18–40 (165 autistic, 146 non-autistic). The survey, developed with input from autistic people, asked about reproductive health conditions, symptoms, knowledge, management, and experiences with healthcare. Results showed that autistic participants reported more reproductive health conditions and symptoms than non-autistic participants. They were more likely to have irregular menstrual cycles, mental health or sensory changes related to their cycle, and difficulties managing their reproductive health. Autistic participants also reported more negative experiences with healthcare overall and in specific areas. These findings suggest that autistic AFAB individuals face a higher reproductive health burden and greater challenges in accessing and managing care. Providing tailored education and adjusting healthcare services to individual needs are important steps toward fair and effective reproductive healthcare for autistic people.

## Introduction

Autism is a common, lifelong neurodevelopmental condition that influences how individuals perceive, process and engage with the world.^1^ Autistic people experience differences and challenges in areas such as social interaction, behavioral and cognitive flexibility, and sensory processing, which can vary widely from person to person.

As awareness and identification of autism have increased,^2^ so too has recognition of the diverse support and healthcare needs across the lifespan.^3–5^Co-occurring conditions, both somatic and psychiatric, are more common in autistic than non-autistic people.^3,5^ Autistic females in particular represent a group with increased risk for different health problems and increased healthcare utilization.^6–8^

Despite this increased healthcare need and risk for various conditions, women and girls have historically been underdiagnosed with autism,^9,10^ partly due to biases in diagnostic criteria and societal expectations around gender-related behavior such as behavioral problems, social communication abilities and relationships.^11^ With the growing recognition and identification of autism in girls and women,^2^ there is increasing awareness of their distinct health needs. Yet, reproductive healthcare remains a significantly underexplored area in both research and clinical practice.

Autistic people assigned female at birth (AFAB) may experience reproductive health problems to a larger extent. Existing evidence points towards higher rates of irregular menstruation, dysmenorrhea, premenstrual disorders (PMDs), polyendocrine metabolic ovarian syndrome (PMOS), as well as menopause symptoms.^12–16^ However, evidence across studies remains inconsistent.^14^ Moreover, despite this increased need for reproductive care, research shows that autistic people make use of obstetric and gynaecological services and screenings to a lesser extent than their non-autistic peers.^17–19^

Autistic people report difficulties and barriers in accessing healthcare that result in unmet healthcare needs.^20–22^ Barriers to care exist on multiple levels, including for individuals (e.g. making appointments, sensory issues), with providers (e.g. communication, limited autism understanding) and systemically (e.g. availability and accessibility, complexities of care chain), increasing the complexity of navigating care.^21,23^ In addition, difficulties in recognising and describing symptoms may further delay help-seeking or lead to uncertainty about when to seek medical attention, compounding these barriers.^24,25^ Importantly, barriers in accessing and navigating healthcare due to communication differences, sensory sensitivities, or previous negative experiences with providers might play a role.^24^ These challenges, combined with limited accessibility and provider understanding, may contribute to poorer overall health outcomes reported among autistic adults.^21,25^

Existing research has primarily focused on general healthcare access and experiences,^25^ with limited attention to specific reproductive health needs of autistic people AFAB.^26^ One UK-based mixed-methods study^22^ focusing on autistic people AFAB reported that reproductive healthcare providers rarely offer sensory and communicative accommodations, and lack awareness of the association between autism and reproductive health. However, the study was restricted to autistic people AFAB and did not include a non-autistic comparison group, which limits the ability to identify healthcare experiences specific to autism in reproductive settings. Moreover, while existing research^21^ has identified substantial barriers to general healthcare access among autistic individuals, evidence on how they understand, manage, and access care for reproductive health concerns remains limited. This lack of evidence limits understanding of both the prevalence and management of reproductive health difficulties, and the quality of reproductive healthcare autistic women receive, thereby constraining efforts to develop accessible, informed, and equitable models of care.

The present study aimed to examine differences in reproductive health and healthcare experiences between autistic and non-autistic individuals assigned female at birth. The study assessed group differences in the prevalence of reproductive health conditions and symptoms, the impact of the menstrual cycle on mental health and sensory sensitivity, and participants’ knowledge and management of their reproductive health. Finally, in line with a study examining healthcare experiences more broadly^25^ we evaluated reproductive healthcare experiences across five domains – system problems, access and advocacy, communication, anxiety, and sensory experiences – to determine whether autistic participants reported greater inequality in care. Together, these analyses provide a comprehensive overview of reproductive health disparities and highlight areas for improving accessibility and equity in reproductive healthcare.

## Methods

### Study design

We conducted a cross-sectional online survey to explore reproductive health and healthcare experiences among autistic and non-autistic people. The survey was created and administered using Qualtrics and required around 30 to 40 minutes to complete, with 71.4% of participants completing it in under 40 minutes. Survey items were developed based on previous research examining healthcare experiences in autistic individuals^25^ and adapted to focus specifically on reproductive health. To ensure relevance and clarity, we sought input from 10 autistic individuals during the survey development phase, who were contacted via internal email lists. Individuals responded to a short feedback survey where they were asked about concerns regarding phrasing, content, clarity, or accessibility to example survey items. Their feedback was used to refine the content and structure of the survey. Following this revision process, the final survey was disseminated through established networks and autism research databases including the Autistica network and the Cambridge Autism Research Database (CARD). To recruit non-autistic individuals as a comparison group, we further advertised the study through social media (Reddit) and later via Prolific. Study advertisements referred to neurodivergence and reproductive health. Data collection took place from April 2024 to July 2025. This manuscript was prepared according to STROBE reporting guidelines for observational studies. The study was approved by the King’s College London ethics committee (HR/DP-23/24-41224).

Participants were eligible to take part if they were: assigned female at birth (AFAB), aged between 18 and 40 years, and identified as either autistic (formally diagnosed or self-identified, reflecting evidence of historical underdiagnosis in females^27^ 5) or non-autistic. At the beginning of the study, participants were presented with an online information sheet outlining the aims of the study. Written informed consent was obtained electronically from all participants. Participants were informed that they could withdraw at any time by closing their browser during the study or by notifying the researchers up to a specified date indicated in the information sheet, after which their data were removed during the data cleaning process. Upon completion, participants were debriefed and links to a range of support services were provided to everyone. Participants were offered to enter a random draw of thirty-five £25 Amazon or Love2shop vouchers. To recruit additional non-autistic participants, we later used Prolific – an online research platform for surveys - where individuals were compensated at an hourly rate of £6. This constituted a separate form of payment from the voucher system used earlier and was used since the initial group.

### Participants

A total of 389 completed surveys were recorded. Out of these, 78 were excluded based on meeting one or more of the following criteria: low completion rate or low quality responses (less than 90% of questions completed or answering the same response to every item, not responding to open text questions, n=38), having a very short completion time (under 7 minutes, n=38), age (over 40 or younger than 18, n=1), or missing values (for sex variable, n=1). In the final sample of 311 participants, the autistic group (n = 165) consisted of 139 individuals who reported an autism diagnosis and 26 individuals that self-identified as autistic (see sensitivity analyses). The remaining participants (n = 146) formed the non-autistic comparison group. The sample was predominantly White, UK-based, and university educated. The mean age of the sample was 30.9 years (SD = 5.9), with a median of 31 and a range of 18-40. Autistic and non-autistic participants were comparable in terms of age and location of residence. Autistic participants, both diagnosed (M = 170.9) and self-identified (M = 171.7) showed higher Comprehensive Autistic Trait Inventory (CATI)^26^ scores, were more likely to report co-occurring attention deficit hyperactivity disorder (ADHD) and other forms of neurodivergence and more likely to identify as gender diverse. In terms of ethnicity and education, autistic participants were more likely to identify as White, less likely to identify as Asian or Black, and more likely to hold foundation/diploma or doctoral-level qualifications than non-autistic participants. See Table 1 and Supplementary Table 1 for demographic characteristics for both groups and Supplementary Figure 1 for a flow diagram summarizing participant inclusion and analytic sample derivation across study analyses.

**Table 1.** Participant demographics.

Characteristics	N	Autistic N = 165^1^	Non-autistic N = 146^1^	p-value^2^
**Mean CATI score (SD)**	304	171.0 (±17.2)	103.9 (±28.7)	<0.001
**ADHD diagnosis**	311	81 (49.1%)	30 (20.5%)	<0.001
**Other Neurodivergence**	311	​	​	<0.001
Any ND	​	76 (46.1%)	10 (6.8%)	​
No ND	​	87 (52.7%)	136 (93.2%)	​
Prefer not to say	​	<5	<5	​
**Age (years)**	311	31.1 (±6.2)	30.6 (±5.5)	0.4
**Gender**	311	​	​	<0.001
Woman or girl	​	142 (86.1%)	145 (99.3%)	​
Man or boy	​	<5	0 (0%)	​
Nonbinary or genderfluid	​	16 (9.7%)	0 (0%)	​
Other	​	< 5	<5	​
**Ethnicity**	311	​	​	0.009
Asian	​	<5	10 (6.8%)	​
Black	​	<5	11 (7.5%)	​
Hispanic or Latinx	​	<5	<5	​
Mixed ethnicities	​	7 (4.2%)	7 (4.8%)	​
White	​	148 (89.7%)	116 (79.5%)	​
Self-described	​	<5	<5	​
Prefer not to say	​	<5	<5	​
**Education**	311	​	​	0.001
Some secondary/high school education	​	9 (5.5%)	8 (5.5%)	​
Secondary/High School Leavers qualifications	​	24 (14.6%)	19 (13.0%)	​
Vocational or Professional Qualifications	​	7 (4.2%)	9 (6.2%)	​
University	​	121 (73.3%)	110 (75.3%)	​
Other	​	<5	0 (0%)	​

SD = Standard deviation.

Values fewer than 5 are reported as ‘<5’ to preserve confidentiality and prevent identification of individual subjects.

^1^n (%).

^2^Wilcoxon rank sum test; Pearson’s Chi-squared test; Fisher’s exact test.

### Materials

#### Demographic questions and autism assessment

Participants first completed demographic questions including sex, gender, ethnicity, country of residence, and highest level of education attained. Location was measured by asking participants to report the country in which they currently reside and was later grouped into UK vs. non-UK for analysis due to the predominance of UK participants. Participants also provided information on autism diagnosis (medically diagnosed and self-identified) as well as ADHD and other neurodivergence. Autistic traits were assessed using CATI.^28^

#### Reproductive health status

Based on a review of the literature and a scoping stage during study development, which identified commonly reported reproductive and endocrine-related conditions and symptoms, we assessed 13 self-reported individual reproductive health conditions (e.g. premenstrual syndrome, and breast, uterine and ovarian cancers/tumors/growth) and 9 reproductive and endocrine-related symptoms (e.g. atypically heavy menstrual bleeding, painful periods, severe acne). Participants were also asked whether they received sufficient information and education on women’s reproductive health from a healthcare provider or whilst in school (knowledge). They also answered questions regarding their confidence in managing their reproductive health adequately and whether they experienced challenges in relation to this (management). Lastly, participants were asked if they noticed changes in their mental health or sensory differences in relation to their menstrual cycle (impact). Composite scores were created for the knowledge, management, and impact constructs. Knowledge and management each consisted of three Likert-scale items, while impact consisted of two items. Responses were recorded on a 5-point Likert scale ranging from strongly agree to strongly disagree. Items were coded so that higher scores reflected greater perceived challenges or impacts related to reproductive health, and reverse coding was applied where necessary to ensure consistent scoring direction. Mean scores across items within each construct were calculated for each participant. Internal consistency in the present sample was moderate for the knowledge scale (Cronbach’s α = .66) and impact scale (α = .64). The management scale showed lower internal consistency (α = .45), which may reflect that the items capture related but distinct aspects of reproductive health management. Autistic participants were further asked if they noticed changes in autistic traits across their cycle.

#### Reproductive healthcare experiences

Questions from a previous study^25^ exploring sensory experiences, communication, anxiety, access and advocacy and system problems in autistic individuals in a general healthcare setting were adapted in this study to be more specifically focused on reproductive health. We included multiple choice questions and free text questions, with reproductive health and healthcare experiences being assessed on a 5-point Likert scale (Strongly agree, Somewhat agree, Neither agree nor disagree, Somewhat disagree, or Strongly disagree).

Please see Supplementary Text 1 for additional information.

### Statistical analysis

All statistical analyses were conducted in R Version 4.5.0. We used a significance threshold of *p* < .05. Given the number of statistical tests across related outcomes, False Discovery Rate (FDR) correction using the Benjamini–Hochberg procedure was applied to control for multiple comparisons.

#### Reproductive health

Associations between autism diagnosis and reproductive health outcomes (individual conditions, symptoms, and composite variables for any condition or symptom) were assessed using binary logistic regressions to calculate odds ratios (OR) with 95% confidence intervals (CIs).

Reproductive health analyses were conducted on the full analytic sample (N = 311), with no variation in sample size across models. Three models were run: (1) crude, (2) adjusted for age, (3) adjusted for age, ADHD diagnosis, and other neurodivergence. Crude (unadjusted) regression models are presented in Supplementary Table 2. Sensitivity analyses excluded self-diagnosed autistic participants (N = 285). FDR correction was applied across all 20 tests of reproductive health outcomes, with results interpreted at *q* < .05. Period regularity responses were collapsed into four categories: Regular, Irregular, Menopause-related, and Other. When multiple categories were selected, a primary category was assigned according to the following priority: Menopause-related > Irregular > Regular > Other. Group comparisons were performed using Fisher’s Exact Test when expected counts were <5, and Chi-squared tests otherwise, with Cramér’s V reported as an effect size. Mean cycle length in days was also calculated for each group and compared using a Wilcoxon rank sum test reported with its effect size.

#### Knowledge, management, and impact

Mean scores for the knowledge, management, and impact constructs were calculated for each participant based on the composite measures described above. Group differences were evaluated using Wilcoxon rank-sum tests, with rank-biserial correlation as effect size. FDR correction was applied across the three group comparison tests to control for multiple testing, with results interpreted at *q* < .05.

#### Healthcare inequality section scores

Healthcare inequality (HIE) was measured across five subcategories (System problems, Access and advocacy, Anxiety, Communication, and Sensory experiences, see Supplementary Table 3). Items within the subcategories with responses indicating negative experiences coded as 1 and positive or neutral experiences as 0. Likert-scale responses were dichotomized to capture the presence versus absence of negative healthcare experiences and to facilitate interpretation of composite scores as the proportion of adverse experiences within each domain. While this approach improves interpretability, it may reduce variability and sensitivity to gradations in responses. HIE composite scores ranging from 0 to 1 were calculated for each subsection as the proportion of negatively experienced items out of the items the participant answered within that subsection, and for the overall score as the proportion across all answered items. Participants with more than one missing response per subsection or overall were excluded from the respective calculations to ensure that subsection scores reflected a sufficient proportion of completed items and were not disproportionately influenced by missing data. In addition, participants who answered “No” to the question “Have you ever spoken to a healthcare provider about your reproductive health (health of the female reproductive system – vagina, uterus, ovaries, and breasts)?” were excluded from this analysis because the HIE items assessed experiences occurring during interactions with healthcare providers, and therefore were not applicable to individuals without such encounters. As a result, HIE analyses were conducted on a subset of participants with available data (N = 235). HIE scores closer to 1 indicate worse healthcare experiences.

Fractional logistic regression models were run to determine whether each subsection of HIE scores or the total HIE score differed based on autism status. Models were conducted both unadjusted and adjusted for age, ethnicity, education, and location. Adjusted models included smaller samples (range N = 178–194) due to missing covariate data. Because the HIE scores represent proportions ranging from 0 to 1, we did not standardize these outcomes. FDR correction was applied across the six tests (the five inequality domains and the total score) to account for multiple comparisons with results interpreted at *q* < .05. Sensitivity analyses (N = 285) excluded self-diagnosed autistic participants, with further variation in sample size in HIE models due to missing data.

Post-hoc power analyses were conducted in G*Power 3.1^29^ based on the obtained sample sizes and observed effect sizes. Adequate to high power was observed for the overarching outcomes (any reproductive health condition or symptom, menstrual-cycle impact, reproductive health knowledge and management, period regularity, Supplementary Table 4). Power was lower for several individual reproductive health conditions and symptoms.

## Results

### Differences in self-reported reproductive health and menstruation

Overall, 44% of autistic participants self-reported at least one reproductive health condition (see Table 2), compared to 28% of non-autistic participants, with premenstrual syndrome (PMS) (22% vs. 12%), premenstrual dysphoric disorder (PMDD) (14% vs. 4%) and polyendocrine metabolic ovarian syndrome (PMOS) (13% vs. 9%) being the most commonly reported. Compared to non-autistic participants, autistic participants showed higher odds for all conditions except hypothyroidism, with statistically significantly higher odds for any reproductive health condition (OR = 1.96 [1.21-3.17], *p* = .006), PMS (OR = 2.10 [1.12-3.94], *p* = .02) and PMDD (OR = 3.77 [1.49-9.56], *p* = .005) in age-adjusted analyses. When additionally adjusting for other neurodivergence, ORs showed the same direction but were attenuated and no longer statistically significant (OR_anycondition_ = 1.55 [0.90-2.67]). Crude models showed a similar pattern of associations, although effect estimates were attenuated following adjustment (Supplementary Table 2).

**Table 2.** Differences in condition and symptom prevalences between autistic and control participants.

​	Age adjusted model				Neurodivergence adjusted model	
	Autistic (n = 165)	Non-autistic(n = 146)	OR [95% CI]	p-value	OR [95% CI]	p-value
**Any condition**	72 (43.6%)	41 (28.1%)	1.96 [1.21-3.17]	< .01	1.55 [0.90-2.67]	.115
Premenstrual syndrome (PMS)	36 (21.8%)	17 (11.6%)	2.10 [1.12-3.94]	< .05	1.79 [0.88-3.62]	.106
Premenstrual dysphoric disorders (PMDD)	23 (13.9%)	6 (4.1%)	3.77 [1.49-9.56]	< .01	2.53 [0.90-7.10]	.078
Polyendocrine metabolic ovarian syndrome (PMOS)	21 (12.7%)	13 (8.9%)	1.47 [0.70-3.08]	.309	1.31 [0.57-3.01]	.532
Endometriosis	10 (6.1%)	6 (4.1%)	1.42 [0.50-4.05]	.516	1.18 [0.36-3.86]	.783
Hypothyroidism	7 (4.2%)	7 (4.8%)	0.82 [0.28-2.43]	.719	0.55 [0.15-2.06]	.377
**Any symptom**	156 (94.5%)	123 (84.2%)	3.23 [1.44-7.25]	< .01	3.08 [1.23-7.71]	< .05
Excessive or atypically heavy menstrual bleeding	118 (71.5%)	78 (53.4%)	2.17 [1.36-3.48]	< .01	2.09 [1.22-3.58]	< .01
Anaemia	114 (69.1%)	77 (52.7%)	1.99 [1.25-3.16]	< .01	1.59 [0.94-2.67]	.083
Unusually painful periods	109 (66.1%)	77 (52.7%)	1.73 [1.09-2.74]	< .05	1.53 [0.91-2.56]	.109
Unusually frequent need to urinate	108 (65.5%)	61 (41.8%)	2.63 [1.66-4.17]	< .001	2.26 [1.35-3.79]	< .01
Atypically increased thirst	90 (54.5%)	34 (23.3%)	3.97 [2.43-6.50]	< .001	3.47 [2.01-5.99]	< .001
Severe acne	69 (41.8%)	44 (30.1%)	1.66 [1.04-2.66]	< .05	1.48 [0.87-2.52]	.145
Hirsutism	66 (40.0%)	38 (26.0%)	1.87 [1.15-3.04]	< .05	1.48 [0.86-2.56]	.161
Hair loss or thinning	62 (37.6%)	45 (30.8%)	1.32 [0.82-2.13]	.254	1.32 [0.77-2.27]	.310
Sudden, unexplained weight loss	23 (13.9%)	19 (13.0%)	1.09 [0.57-2.09]	.799	1.34 [0.65-2.75]	.427

*Note.* Individual analyses for the following conditions were not performed due to small subgroup sizes: Anovulation, Breast cancer/tumors/growths, Delayed puberty, Hyperthyroidism, Hypogonadism, Ovarian cancer/tumors/growths, Precocious puberty, Uterine cancer/tumors/growths.

*OR* odds ratio, *95% CI* 95% confidence internal*, Sig.* significance level.

*P*-value: < .05 = *; < .01 = **; < .001 = ***.

Over 94% of autistic participants reported at least one reproductive health symptom compared to 84% of non-autistic individuals; heavy menstrual bleeding (72% vs. 53%), anaemia (69% vs. 53%) and dysmenorrhea (66% vs. 53%) were the most reported symptoms. Overall, autistic participants showed statistically significantly increased odds for any symptom (OR = 3.23 [1.44-7.25]) and across most individual symptoms (OR_range_ = 1.73 [1.44-7.25] – 3.97 [2.43-6.50], *p* <.05). Similar to reproductive health conditions, additional adjustment for ADHD and other forms of neurodivergence reduced the estimates to some extent, although the attenuation was incomplete for most outcomes (OR_anysymptom_ = 3.08 [1.23-7.71]). However, some associations were no longer statistically significant. Crude models showed a similar pattern of associations, although effect estimates were attenuated following adjustment (Supplementary Table 2).

After applying FDR correction across all 20 reproductive health outcomes, associations with PMS (OR = 2.10 [1.12-3.94], *q* < 0.05), PMDD (OR = 3.77 [1.49-9.56], *q* < 0.05), heavy menstrual bleeding (OR = 2.17 [1.36-3.48], *q* < 0.01), increased thirst (OR = 3.97 [2.43-6.50], *q* < 0.001), and frequent urination (OR = 2.63 [1.66-4.17], *q* < 0.001) remained statistically significant. Sensitivity analyses excluding self-identified autistic participants produced a similar pattern of results, with the direction and magnitude of effects largely unchanged.

**Figure 1.**
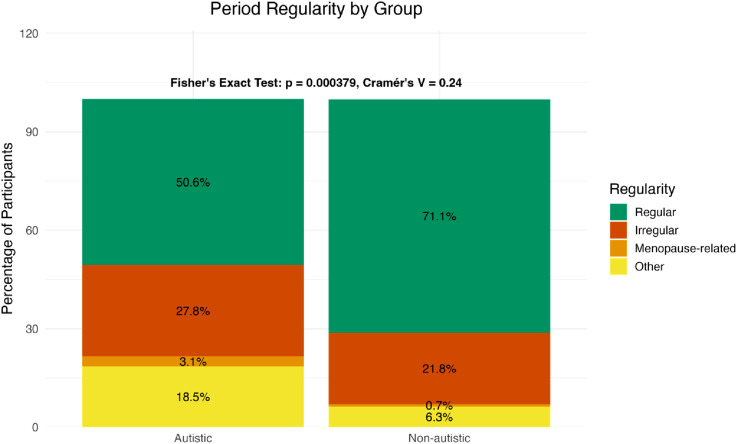
Period regularity by group.

Autistic participants were significantly less likely than non-autistic participants to report regular periods (51% vs. 71%, Fisher’s Exact Test, *p* = 0.0004, Cramer’s V = 0.24, Figure 1). However, no significant group differences were observed in average menstrual cycle length (31 vs 30 days, *p* = 0.202, Supplementary Figure 2).

### Impact of the menstrual cycle

**Figure 2.**
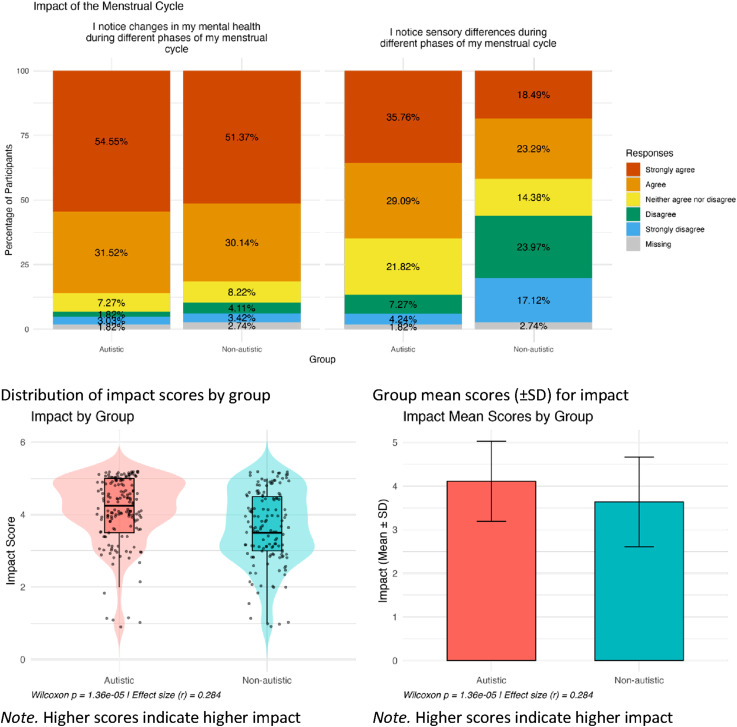
Impact of menstrual cycle on mental health and sensory differences.

**Figure 3.**
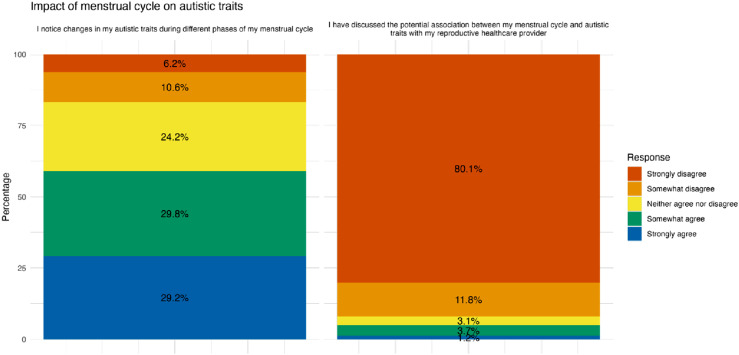
Impact of menstrual cycle on autistic traits.

The mean impact scores combining the influence on mental health and sensory differences were significantly higher for autistic participants (*M* = 4.11, *SD* = 0.92) than for non-autistic participants (*M* = 3.64, *SD* = 1.03, W = 14770, *p* < .001, rank-biserial correlation = .28, 95% CI [.16, .40]) including after FDR correction (*q* < 0.001). Among autistic participants, 86% reported changes in their mental health during different phases of their menstrual cycle (Figure 2) compared to 82% of non-autistic participants. Sensory differences attributed to the menstrual cycle were reported in 65% of autistic and 42% of non-autistic participants. Among autistic participants, 59% reported changes in their autistic traits during different phases of their menstrual cycle. At the same time the majority (92%) of autistic participants reported not having discussed this with their healthcare professional (Figure 3).

### Knowledge and management of reproductive health

**Figure 4.**
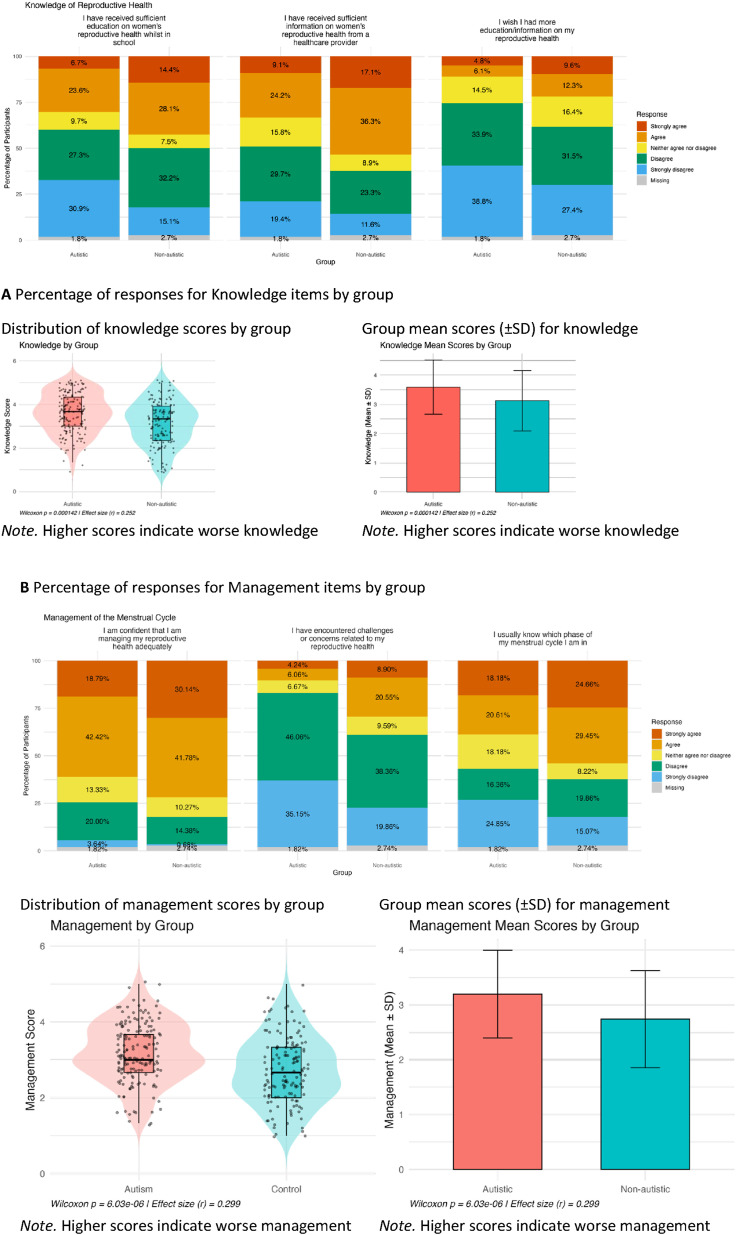
Knowledge and management.

Mean knowledge and management scores were both significantly higher – i.e. worse – for autistic participants (Knowledge: *M* = 3.59, *SD* = 0.92; Management: *M* = 3.20, *SD* = 0.80; W_knowledge_ = 14397, *p* < .001, rank-biserial correlation = .25, 95% CI [.13, .37]) than non-autistic participants (Knowledge: *M* = 3.12, *SD* = 1.03; Management: *M* = 2.74, *SD* = 0.89; W_management_ = 14940, *p* < .001, rank-biserial correlation = .30, 95% CI [.18, .41]). Group differences were significant even after FDR correction (all *q* < 0.001). A higher percentage of autistic than non-autistic participants reported not having received sufficient information on women’s reproductive health from a healthcare provider (49% vs. 35%) or while in school (58% vs. 47%) and wished they had more information or education on their reproductive health (73% vs. 59%, Figure 4(a)). In terms of managing their reproductive health 39% of autistic participants (vs. 54% of non-autistic participants) reported that they are usually aware of the phase in their menstrual cycle they are in (Figure 4(b)). A total of 61% (vs. 72%) further reported being confident in managing their reproductive health. At the same time, 81% of autistic participants (vs. 58%) reported having encountered challenges or concerns in relation to managing their reproductive health.

### Reproductive healthcare experiences

**Figure 5.**
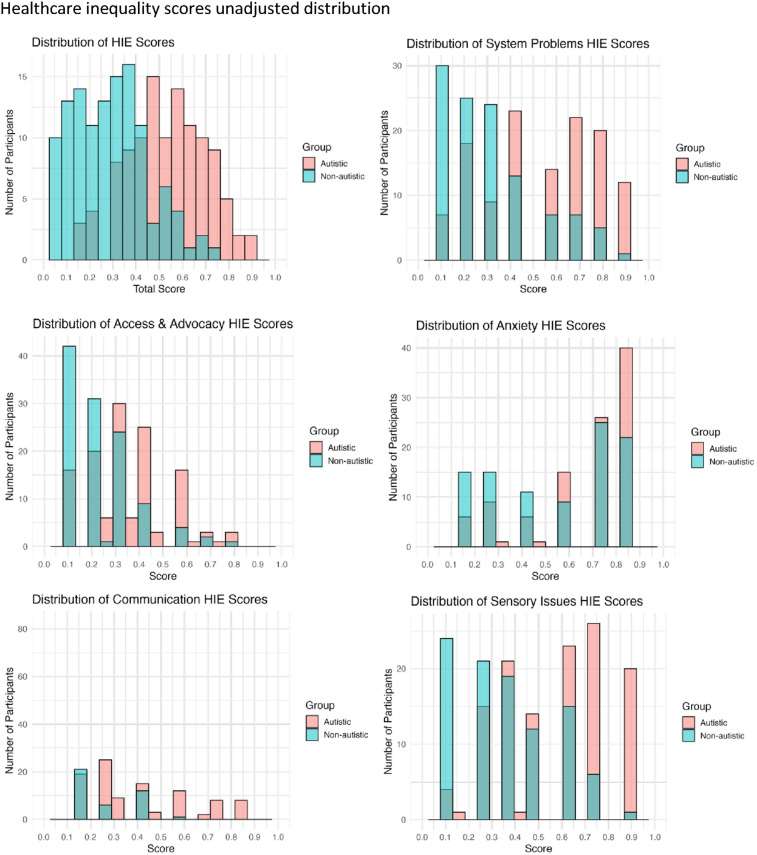
Healthcare inequality scores unadjusted distribution. *Note.* This figure shows the distribution of health inequality (HIE) scores for the overall score and specific domains for autistic and non-autistic participants. Negative healthcare experiences were coded as 1 while neutral or positive experiences were coded as 0. Composite scores ranging from 0 to 1 were calculated based on the proportion of negatively experienced items out of all answered items.

Needing reproductive healthcare but not seeking help was reported by 37% of autistic participants compared with 15% of non-autistic participants. Figure 5 shows the distributions of the unadjusted health inequality scores (HIE) for all participants for the total score and different domains. Autistic individuals consistently showed higher scores indicating healthcare inequalities compared to non-autistic participants. Logistic regression results (Table 3) suggest that higher HIE scores were strongly associated with autism diagnosis. In unadjusted models, autism diagnosis was associated with both higher overall (OR = 2.86 [2.37-3.45]) and subcategory (OR_range_ = 1.81 [1.49-2.22] – 5.31 [3.56-8.13]) HIE scores. Because the HIE outcomes represent proportions ranging from 0 to 1, these ORs reflect the relative likelihood that autistic participants report a higher proportion of negative healthcare experiences within each domain compared with non-autistic participants. After adjusting for age, ethnicity, education, and location, all associations remained statistically significant, although ORs were slightly attenuated. In both adjusted and unadjusted models, all five healthcare inequality domains and the total composite score remained highly significant after FDR correction (all *q* < 0.001). Sensitivity analyses excluding self-identified autistic participants produced the same overall pattern of results, with all significant associations retained and effect estimates remaining similar in magnitude.

**Table 3.** Comparison of autistic and non-autistic individuals health inequality scores.

​	Unadjusted model^a^		Adjusted model^b^	
	OR (95% CI)	p-value	OR (95% CI)	p-value
System problems score	2.92 (2.26, 3.79)	<.001	2.23 (1.60, 3.10)	<.001
Access & advocacy section score	1.81 (1.49, 2.22)	<.001	1.56 (1.21, 2.03)	<.001
Anxiety section score	2.70 (1.97, 3.71)	<.001	2.11 (1.38, 3.25)	<.001
Communication section score	5.31 (3.56, 8.13)	<.001	5.01 (2.94, 8.85)	<.001
Sensory sensitivity section score	3.33 (2.55, 4.36)	<.001	2.96 (2.11, 4.20)	<.001
Total final score	2.86 (2.37, 3.45)	<.001	2.39 (1.88, 3.06)	<.001

*OR* odds ratio, *95% CI* 95% confidence internal*, Sig.* significance level.

*P*-value: < .05 = *; < .01 = **; < .001 = ***.

^a^Fractional Logistic Regression (unadjusted).

^b^Fractional Logistic Regression adjusting for age, ethnicity, education, and location of residence.

## Discussion

Overall, our findings indicate that autistic people AFAB experience a greater burden of reproductive health problems alongside more negative and unequal healthcare experiences than their non-autistic counterparts. Autistic participants were more likely to report at least one reproductive health condition, with higher rates of self-reported PMS and PMDD, as well as a broader range of symptoms including heavy menstrual bleeding, anaemia, and dysmenorrhea. Among those reporting on their menstrual cycle, autistic participants described greater fluctuations in mental health and sensory differences across the cycle than non-autistic participants. Autistic participants further noted changes in autistic traits during different phases. Despite these increased health needs, autistic participants reported lower levels of understanding and less effective management of their reproductive health, alongside a stronger desire for accessible information and guidance. Finally, their reproductive healthcare experiences reflected persistent disparities, with significantly higher healthcare inequality scores across all domains – system problems, access and advocacy, communication, anxiety, and sensory experiences – highlighting widespread barriers that limit autistic individual’s ability to access, navigate, and benefit from reproductive healthcare comparable to their non-autistic peers.

### Reproductive health conditions and symptoms

Autistic people AFAB were more likely to report reproductive health conditions (44% vs. 28%) and symptoms (95% vs. 84%) compared to non-autistic people AFAB. Specifically, autistic participants AFAB reported higher rates of dysmenorrhea, heavy menstrual bleeding, anemia, PMS and PMDD compared to non-autistic participants. Our study finds similar or higher percentages of reproductive health conditions to those reported in a cross-sectional England-based survey including over 59,000 individuals AFAB,^30^ likely reflecting differences in how conditions are defined and assessed, as well as variation in sample characteristics. In terms of symptoms, the elevated frequency of menstrual pain aligns with prior evidence that autistic people AFAB experience heightened dysmenorrhea and increased sensitivity to cycle-related pain, potentially linked to overlapping sensory sensitivities.^31^ Heavy menstrual bleeding and (potentially related) anemia may reflect the cumulative effects of menstrual irregularities and uterine pathology, which appear more common in autistic populations AFAB.^16^ Consistent with this, prior research has identified increased rates of reproductive-system diagnoses and gynecologic symptoms - including menorrhagia and irregular cycles - among autistic women.^16^ The higher prevalence of self-reported PMS in this sample also supports earlier findings that autistic individuals may experience more severe premenstrual mood and physical symptoms,^32^ although evidence for PMDD remains mixed, possibly reflecting heterogeneity in assessment methods and demographic differences between samples.^14^ Moreover, autism has been linked with androgen related conditions such as PMOS, hirsutism, and severe acne.^15,33^ Large registry-based analyses have further shown bidirectional associations between autism and PMOS as well as autism and endometriosis, suggesting shared biological mechanisms.^34–36^ Taken together, this pattern indicates that hormonal, sensory, and inflammatory processes – along with elevated baseline rates of reproductive-system diagnoses – likely interact to produce the greater burden of menstrual and gynecological conditions observed in autistic participants AFAB.

### Reproductive health management and healthcare inequalities

Autistic participants reported lower reproductive-health knowledge and greater difficulties managing menstrual and gynecological symptoms than their non-autistic peers, reflecting a well-documented gap in both education and clinical support for reproductive self-care.^22,37^ Qualitative and mixed methods research consistently shows that autistic people receive less comprehensive or less accessible education about puberty, menstruation, and sexual health, often because healthcare providers, educators, or caregivers underestimate their reproductive needs or assume disinterest in sexuality and relationships.^37–40^ Autistic individuals themselves describe that their needs for relationships and sexual expression are not recognized and report experiences of infantilization in this context.^40,41^ Stereotypes and assumptions about autistic people’s sexual health can delay access to appropriate information, contributing to uncertainty, distress, and reduced autonomy during puberty and adulthood. Studies also highlight that autistic people who menstruate frequently report needing individualised education formats and sensory-informed strategies for pain and hygiene management, yet these accommodations are rarely offered.^37^ Tailored education formats that account for autistic people's sensory, communication, and cognitive needs is necessary to close gaps in sexual and reproductive health knowledge.^41^

Moreover, autistic people often describe challenges that likely impact gynecologic care, including limited provider knowledge of autism, difficulties communicating symptoms, and lack of continuity in care planning.^22,25^ Collectively, this evidence suggests that the gaps in reproductive-health knowledge and management observed here are rooted not only in individual educational barriers but also in systemic patterns that fail to recognize autistic people’s healthcare and adjustment needs.

These gaps in education and information contribute to broader healthcare inequities that affect autistic people’s access to mental and physical healthcare services,^24,42^ the quality of care they receive, and their overall reproductive health outcomes. This pattern was reflected in the higher overall and domain-specific HIE scores reported by autistic people AFAB in our study compared to non-autistic people AFAB. Differences were evident across multiple domains including access and advocacy, communication, anxiety, system problems and sensory experiences, spanning individual, provider and systemic levels of care.

At the individual level patterns of care-seeking emerged as a contributor to healthcare access disparities, in line with previous findings for general practitioner visits.^21^ Twice as many autistic compared to non-autistic people AFAB reported needing care but choosing not to seek it. Practical barriers resulting from the interaction with healthcare providers such as scheduling appointments, traveling to healthcare facilities, and managing anxiety and sensory discomfort associated with visits further increase healthcare inequities, contributing to delayed or emergency-only healthcare utilization. Moreover, in line with previous research,^22^ difficulties in communication were reported including explaining their symptoms, initiating conversations about reproductive health concerns when not prompted, asking questions, or understanding what their healthcare provider means when discussing reproductive health. Together with previous findings noting difficulties communicating with their provider and limited provider knowledge and awareness around how autism may affect reproductive health^22^ and healthcare access in general,^21^ our findings further highlight the need for healthcare professionals to strengthen their knowledge and skills around autism-aware care and tailoring their communication. At the system level, barriers included inadequate availability of services, limited support and guidance following reproductive health diagnoses, and insufficient consultation time to accommodate different communication styles and processing needs. These systemic shortcomings perpetuate inequities by making reproductive healthcare less accessible, responsive, and inclusive for autistic individuals.

While the experiences reported in our study mirror established patterns in autistic people's interactions with the healthcare system,^22–24,42^ they demonstrate how these challenges manifest within gynecologic care, a context that has been largely overlooked in prior research. Collectively, these barriers contribute to unmet healthcare needs and increase the risk of adverse reproductive health outcomes among autistic people AFAB. Negative experiences with reproductive healthcare, in turn, can reinforce avoidance and underutilization of services.^17^ Addressing these inequities requires coordinated strategies that target all levels,^43^ and are developed in partnership with autistic people to ensure that reproductive healthcare is both equitable and accessible. Key adjustments for mental and physical healthcare previously identified by autistic individuals concern the sensory environment (e.g. noise, light etc.), clinical and service context (e.g. appointment length and format, support before appointments etc.), and clinician knowledge and communication (e.g. autism knowledge, familiarity with the clinician, information summary etc.).^44^ Despite being rated as important, these adjustments were reported to be rarely implemented or available.^44^ Implementing appropriate adjustments and improving access to high-quality, autism-informed reproductive healthcare is not only essential for reducing health disparities but also for enhancing overall quality of life and wellbeing among autistic people AFAB.^45^

### Limitations

While this is one of the first studies comparing reproductive healthcare experiences among autistic and non-autistic individuals assigned female at birth, several limitations should be considered when interpreting the findings. First, autism diagnoses were self-reported and we included individuals that self-identified as autistic (although see sensitivity analyses), which may introduce misclassification bias. Without access to clinical confirmation, we cannot verify diagnostic accuracy. However, participants in the autism group, both diagnosed and self-identified, scored significantly higher on the CATI, providing some support for valid group differentiation. Nevertheless, reliance on self-reported diagnoses means that some individuals may have been incorrectly classified, which could influence the strength of the observed associations. Second, the study relied on self-perceived and self-reported experiences, which may be influenced by individual interpretation, recall, and awareness of healthcare norms. Differences observed between groups may therefore reflect both true disparities in experiences and variations in perception or reporting style. However, previous research indicates that self-reported information on reproductive events is generally recalled with reasonable accuracy.^46^ Therefore, while self-report may introduce some measurement error, it is unlikely to fully account for the differences observed between autistic and non-autistic participants unless reporting accuracy systematically differs between groups. The findings may instead reflect a combination of true differences in experiences and potential variations in how experiences are perceived or reported. Moreover, given that obstetric and gynaecological services are used less by autistic people, reliance on diagnosed conditions might underestimate the true association. Third, the sample was recruited through a convenience, online, cross-sectional design. This approach may have introduced selection bias, particularly as individuals with internet access, higher literacy levels, and the capacity to complete a lengthy survey were more likely to participate. This is also reflected in the high education background observed across the autistic and non-autistic groups. Consequently, autistic individuals with moderate to severe intellectual disability, as well as those with lower support needs but less engagement in online communities, are likely underrepresented. Recruitment through online platforms and autism-related networks may also have resulted in a sample that is more engaged with autism-related topics or healthcare issues than the broader population. Fourth, the sample was predominantly white and UK-based, which limits the generalizability of the findings to more diverse or non-UK populations. In the UK, healthcare is provided through a universal system that is free at the point of use. This context may influence both access to and experiences of care, and findings may not directly translate to healthcare systems with different structures or costs. In addition, recruitment through autism-specific databases and networks may have led to an overrepresentation of individuals with a strong interest in autism or healthcare advocacy. Similarly, non-autistic participants might have been drawn to the study due to a personal or professional interest in autism. Moreover, individuals, both autistic and non-autistic with relatively poorer reproductive health and negative healthcare experiences might have been more likely to participate in this study, influencing our findings. Finally, the cross-sectional design prevents any inference about causality or directionality between autism status, healthcare experiences, and reproductive health outcomes. Furthermore, the relatively small sample size limited the statistical power of some analyses. A formal a priori power analysis was not conducted, which limits the ability to determine whether the study was adequately powered to detect all expected effects. Post hoc power calculations indicated variation in statistical power across outcomes, with some analyses demonstrating adequate power (>80%) while others, particularly for rarer outcomes, showed low power (as low as 14%). Consequently, the study may have been underpowered to detect some true associations, especially for infrequent outcomes and subgroup analyses where the number of observations was limited. As a result, some potentially meaningful patterns may not have been identified. Future studies using larger population-based datasets would provide greater statistical power to examine rare outcomes and subgroup differences more robustly and to validate the findings reported here.

## Conclusion

Autistic individuals experience substantially higher rates of reproductive health conditions and symptoms, greater menstrual-related impacts on mental health and sensory experiences, and more challenges in managing their reproductive health compared to non-autistic individuals. At the same time, they face persistent disparities in healthcare arising from the interplay of individual, provider and systemic factors. To reduce these inequities, reproductive healthcare must become more accessible, inclusive and responsive to the diverse needs of autistic people AFAB. This includes improving accessibility, communication and creating environments that accommodate sensory differences and anxiety around healthcare. Equally important is strengthening education and support for people AFAB to facilitate informed reproductive health management. Together, these efforts are essential steps toward achieving truly equitable reproductive healthcare where autistic people AFAB are recognized, understood, and supported in their own care.

## Supplemental material

Supplemental material - Reproductive health and healthcare experiences in autistic and non-autistic individuals assigned female at birth Supplemental material for Reproductive health and healthcare experiences in autistic and non-autistic individuals assigned female at birth by Lili Bokor, Francesca Happé, Elizabeth Weir, Donghao Lu, Gavin R. Stewart, Miriam I. Martini, Mark J. Taylor in Women’s Health.

## Data Availability

Due to the confidentiality and data protection requirements agreed upon in the approved protocol, the datasets generated and analysed during the current study cannot be made publicly available. De-identified data may be made available to qualified researchers upon reasonable request, subject to a data sharing agreement and approval from the relevant King’s College London governance bodies. Requests for access should be directed to the corresponding author.*
