# Supplementary material for: Reproductive health and healthcare experiences in autistic and non-autistic individuals assigned female at birth
Source: Womens Health (Lond). 2026 Jul 7;22:17455057261465645. doi: 10.1177/17455057261465645 (PMC13342387; doi:10.1177/17455057261465645)
Supplement: Supplemental material - Reproductive health and healthcare experiences in autistic and non-autistic individuals assigned female at birth [file sj-pdf-1-whe-10.1177_17455057261465645.pdf]

Supplementary information

Supplementary Text 1

Participant demographic characteristics were summarised by group using means and standard deviations for continuous variables and frequencies with percentages for categorical variables. ADHD diagnosis was coded as Yes/No, including both clinical and self-diagnosed participants, and other neurodivergence was collapsed into "Any neurodivergence," "No neurodivergence," or "Prefer not to say." The total CATI score was computed by summing responses across the 42 items (each scored 1–5), with five items reverse-coded so that higher values consistently indicated greater difficulties. Participants with missing data on any CATI item were excluded from the total score calculation. Demographic and CATI variables were compared between groups using chi-square tests for categorical variables and t-tests for continuous variables. Survey items assessing reproductive health knowledge, management, and impact on mental health, sensory experiences and autistic traits were reverse-coded where necessary.

Supplementary Figure 1 - Flow diagram of participants and analytic samples across study analyses

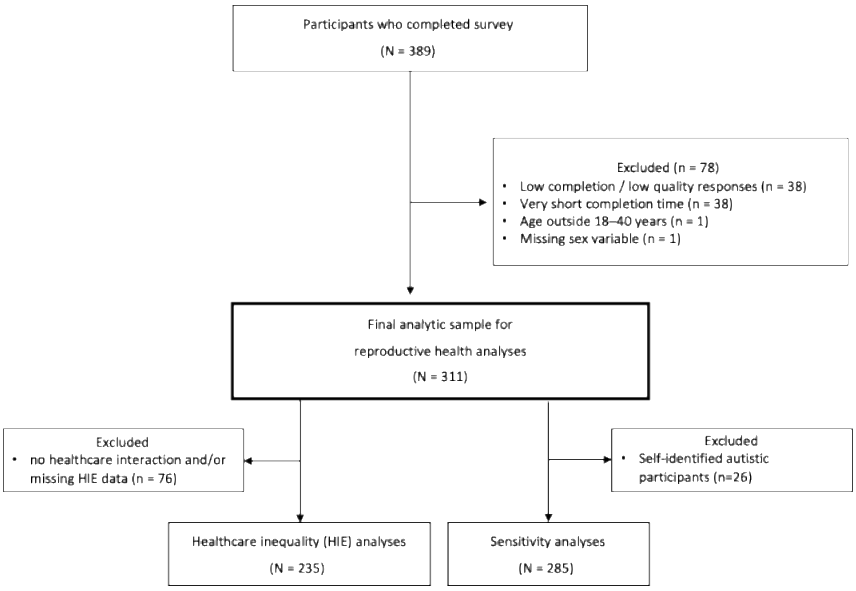

Note. The flowchart shows sample sizes for the main and sub-analyses.

Supplementary Figure 2 - Cycle length by group

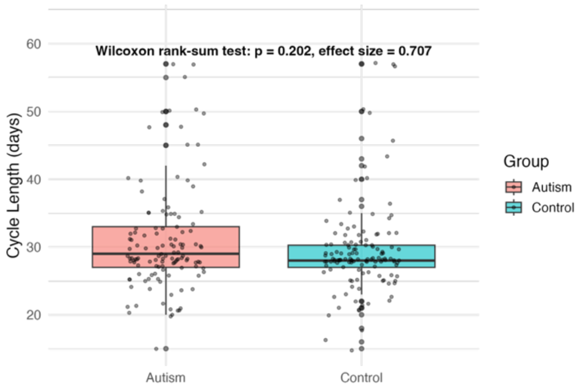

Supplementary Table 1

**Characteristics**	**N**	**Autism** N = 165[1]	**Control** N = 146[1]	**p-value**[2]
**Location of residence**	231			0.12
South East England		24 (14.5%)	14 (9.6%)	
London		11 (6.7%)	21 (14.4%)	
North West England		16 (9.7%)	16 (11.0%)	
East of England		10 (6.1%)	9 (6.2%)	
West Midlands (England)		8 (4.8%)	10 (6.8%)	
South West (England)		12 (7.3%)	12 (8.2%)	
Yorkshire and the Humber (England)		5 (3.0%)	<5	
East Midlands (England)		8 (4.8%)	8 (5.5%)	
North East and Cumbria (England)		6 (3.6%)	<5	
Scotland		<5	10 (6.8%)	
Wales		5 (3.0%)	9 (6.2%)	
Northern Ireland		<5	<5	
Outside of UK		<5	<5	

Note. Counts <5 suppressed to avoid identification

Supplementary Table 2 - Crude (unadjusted) associations between autism status and reproductive health outcomes

	Crude model			
	**Autism (n = 165)**	**Control (n = 146)**	**OR [95% CI]**	**p-value**
**Any condition**	72 (43.6%)	41 (28.1%)	1.98 [1.23–3.19]	.005
Pre-menstrual syndrome (PMS)	36 (21.8%)	17 (11.6%)	2.12 [1.13–3.97]	.019
Pre-menstrual dysphoric disorder (PMDD)	23 (13.9%)	6 (4.1%)	3.81 [1.50–9.64]	.005
Polyendocrine metabolic ovarian syndrome (PMOS)	21 (12.7%)	13 (8.9%)	1.51 [0.73–3.14]	.270
Endometriosis	10 (6.1%)	6 (4.1%)	1.50 [0.53–4.24]	.443
Hypothyroidism	7 (4.2%)	7 (4.8%)	0.88 [0.30–2.58]	.819
**Any symptom**	156 (94.5%)	123 (84.2%)	3.24 [1.45–7.26]	.004
Excessive or atypically heavy menstrual bleeding	118 (71.5%)	78 (53.4%)	1.89 [1.17–3.07]	.010
Anaemia	114 (69.1%)	77 (52.7%)	2.19 [1.37–3.50]	.001
Unusually painful periods	109 (66.1%)	77 (52.7%)	2.00 [1.26–3.18]	.003
Unusually frequent need to urinate	108 (65.5%)	61 (41.8%)	2.64 [1.67–4.18]	<.001
Atypically increased thirst	90 (54.5%)	34 (23.3%)	3.95 [2.42–6.46]	<.001
Severe acne	69 (41.8%)	44 (30.1%)	1.35 [0.84–2.17]	.211
Hirsutism	66 (40.0%)	38 (26.0%)	1.67 [1.04–2.67]	.033
Hair loss or thinning	62 (37.6%)	45 (30.8%)	1.08 [0.56–2.08]	.812
Sudden, unexplained weight loss	23 (13.9%)	19 (13.0%)	1.74 [1.10–2.76]	.017

Note.
Individual analyses for the following conditions were not performed due to small subgroup sizes: Anovulation, Breast cancer/tumors /growths, Delayed puberty, Hyperthyroidism, Hypogonadism, Ovarian cancer/ tumors/ growths, Precocious puberty, Uterine cancer/tumors/growths

OR odds ratio, *95% CI* 95% confidence internal, *Sig.* significance level
P-value: < .05 = * ; < .01 = ** ; < .001 = ***

Supplementary Table 3 - Healthcare inequality items

Domains	Survey questions
**System problems**	Are you able to see a healthcare provider (gynaecologist, GP, nurse practitioner) about your reproductive health as often as you would like?
	When you go in to see a healthcare provider (gynaecologist, GP, nurse practitioner) about your reproductive health, are you able to see the same person each time?
	Please indicate to which extent you agree with the following statements with regard to making appointments with your reproductive healthcare provider.<br>• *If I know that it will be difficult to make an appointment I will postpone getting an appointment*<br>• *In most appointments I have enough time to discuss my concerns with my reproductive healthcare provider*<br>• *I usually leave my appointments knowing what the next steps are (i.e. follow-up appointments, medications, etc.)*<br>• *I am provided with appropriate support after I receive a diagnosis of any kind*<br>• *I think that the reproductive healthcare provider cares about my wellbeing*<br>• *There were times when I did not receive enough support from my reproductive healthcare provider*<br>• *I usually feel like my healthcare provider takes me and my health concerns seriously*
**Access and advocacy**	Has there ever been a time when you needed gynaecological care but did not seek it?
	Please indicate to which extent you agree with the following statements with regard to making appointments with your reproductive healthcare provider.<br>• *I know how to contact my healthcare provider*<br>• *I am able to make appointments for myself*<br>• *I find it easy to make an appointment if I can book online*<br>• *I find it easy to make an appointment by telephone*<br>• *I will wait until it is an emergency before I go to see a reproductive healthcare provider*<br>• *If I need to go to see a reproductive healthcare provider, I am able to get there*<br>• *I usually bring someone along to help support me in my appointments*
	Please indicate to which extent you agree with the following statements with regard to your reproductive healthcare experience.<br>• *I am able to follow a procedure for next steps if asked (for example, I will attend follow-up appointments, annual checkups if applicable, etc.)*
**Anxiety**	Please indicate to which extent you agree with the following statements with regard to making appointments with your reproductive healthcare provider.<br>• *The process of setting up an appointment makes me anxious*
	Please indicate to which extent you agree with the following statements with regard to reproductive healthcare experience.<br>• *The idea of going to see a healthcare professional about my reproductive health makes me feel anxious*<br>• *I feel anxious when I see a different healthcare professional to whom I expected*

	• *The environment of the waiting room makes me feel anxious*<br>• *The environment of the examination room makes me feel anxious*<br>• *The process of picking up a prescription makes me anxious*<br>• *I frequently leave my healthcare provider feeling as though I did not receive any help at all with my reproductive health*
**Communication**	Please indicate to which extent you agree with the following statements with regard to your reproductive healthcare experience.<br>• *I am usually able to explain what my symptoms are*<br>• *I usually ask all the questions I would like to*<br>• *I usually understand what my healthcare provider means when they discuss my reproductive health*<br>• *I usually understand the questions my healthcare provider asks*<br>• *I can bring up a reproductive health concern even if my healthcare provider doesn't ask about it*<br>• *My healthcare provider makes sure I understand the things I need to do to take care of my reproductive health*<br>• *I feel like my healthcare provider understands what I am trying to communicate about my reproductive health*
**Sensory experiences**	Please indicate to which extent you agree with the following statements regarding your gynaecological healthcare experience.<br>• *I am able to describe how my symptoms feel in my body*<br>• *The sensory environment of the waiting room is overwhelming*<br>• *The sensory environment of the examination room is overwhelming*<br>• *My senses frequently overwhelm me which impacts my experience of seeking healthcare / accessing healthcare appointments*<br>• *My senses frequently overwhelm me so that I have trouble focusing on conversations with healthcare professionals*<br>• *I am comfortable with physical touch during gynaecological examinations*<br>• *I experience discomfort during examinations due to the equipment (e.g. the seat, speculum)*<br>• *I am comfortable with the room temperature during gynaecological examinations*

Supplementary Table 4 - Post-hoc power analyses based on observed effect sizes and sample sizes

**Outcome**	**Effect Size**	**Achieved Power (1−β)**
Reproductive health conditions and symptoms		
Any condition	OR = 1.96	0.816
Premenstrual syndrome (PMS)	OR = 2.10	0.880
Premenstrual dysphoric disorder (PMDD)	OR = 3.77	1.000
Polyendocrine metabolic ovarian syndrome (PMOS)	OR = 1.47	0.386
Endometriosis	OR = 1.42	0.331
Hypothyroidism	OR = 0.82	0.141
Any symptom	OR = 3.23	0.998
Excessive/atypically heavy menstrual bleeding	OR = 2.17	0.904
Anaemia	OR = 1.99	0.832
Unusually painful periods	OR = 1.73	0.652
Unusually frequent need to urinate	OR = 2.63	0.980
Atypically increased thirst	OR = 3.97	1.000
Severe acne	OR = 1.66	0.587
Hirsutism	OR = 1.87	0.761
Hair loss or thinning	OR = 1.32	0.227
Sudden unexplained weight loss	OR = 1.09	0.067
Knowledge, management and impact		
Impact	r = .28 (d = 0.58)	0.999
Knowledge	r = .25 (d = 0.52)	0.995
Management	r = .30 (d = 0.63)	1.000
Menstrual cycle characteristics		
Period regularity	Cramér's V = 0.24	0.941
